# Psychological Assessment and Treatment Effectiveness in Mastalgia: Developing a Treatment Algorithm

**DOI:** 10.7759/cureus.46838

**Published:** 2023-10-11

**Authors:** Shubhajeet Roy, Gitika N Singh, Nikhil Verma, Gunjan Parasher, Parijat Suryavanshi

**Affiliations:** 1 Faculty of Medical Sciences, King George's Medical University, Lucknow, IND; 2 General Surgery, King George's Medical University, Lucknow, IND

**Keywords:** danazol, tamoxifen, non-steroidal anti-inflammatory drug, evening primrose oil, treatment options, psychological assessment, mastalgia

## Abstract

Background

Mastalgia often impairs the physical, social, and sexual lives of women. It may manifest in both cyclical or acyclical patterns. The psychoneurotic association of mastalgia has been claimed for a long time in various available literature. Several treatment options have been used and are available in the market for mastalgia, but no specific guidelines are currently in place at the global or local levels. This study aims to evaluate the psychological status and effectiveness of various treatment options in women presenting with mastalgia.

Methods

This study was conducted in the General Surgery outpatient department from February 1 to November 30, 2021, at King George’s Medical University, Lucknow, India. Females of all age groups presenting to the General Surgery outpatient department with unilateral/bilateral breast pain and/or chest wall pain were considered for this study. Pregnant patients, those with a history of allergy to drugs, or those who were lost to follow-up were excluded from the study. The psychological status of patients was assessed using the Depression Anxiety and Stress Scale (DASS-42) scale. Pain assessment was performed using a visual analog scale (VAS). Patients were divided into five categories: (i) isolated chest wall pain, (ii) isolated breast pain, (iii) both chest wall and breast pain, (iv) pain with an associated lump(s), and (v) pain and tenderness isolated over the lump, and two groups: Group-A: VAS≤4, and Group-B: VAS>4. Group B patients in Category iv were randomized into two groups: topical non-steroidal anti-inflammatory drugs (NSAIDs) or evening primrose oil+vitamin E. The next line of treatment was tamoxifen 10mg followed by danazol 100mg followed by ormeloxifene 30mg.

Results

The mean age of 106 participants enrolled was 31.59±10.52 years. The mean scores, using the DASS-42 scale, for depression, anxiety, and stress were 7.31±8.53, 7.08±6.57, and 11.15±8.07, respectively. The depression, anxiety, and stress scores had no significant correlation with pain scores (p =0.84, 0.99, and 0.97 for depression, anxiety, and stress, respectively), or duration (p=0.69, 0.66, and 0.85 for depression, anxiety, and stress, respectively). Twenty-nine of 43 patients (67.44%) responded to topical NSAIDs as first-line treatment, and out of the remaining, 6.98% responded to evening primrose oil + vitamin E, 18.60% to tamoxifen, and 4.65% to danazol. Twenty-nine of 32 patients (90.63%) responded to evening primrose oil+vitamin E as first-line treatment, while 6.25% and 3.12% responded to tamoxifen and danazol, respectively.

Conclusions

Both topical NSAIDs and evening primrose oil + vitamin E were found effective first-line treatment options in the majority of patients. Hence, it is always advisable to start such patients on topical NSAIDs, or evening primrose oil + vitamin E, before switching over (if no resolution of pain is reported with these drugs) to higher and more severe treatment options. The duration or severity of pain did not correlate with the psychological condition of the patient.

## Introduction

Mastalgia or breast pain is a common problem affecting women mostly in the age group of 30-50 years. Its prevalence is highly variable ranging from 41% to 79% [1,2]. Breast pain may sometimes be severe enough to impair their physical, social, and sexual lives. Thus, it is important for affected women to seek medical help, which may also include the fear of breast cancer [3,4]. Mastalgia may be cyclical (associated with the menstrual cycle) or acyclical. Chest wall pain and non-chest wall pain (costochondritis, rib pain, or neck pain referred to as chest wall) can also mimic mastalgia. The stimulatory effect of estrogen on breast tissue, and lower progesterone levels, may contribute to mastalgia. Hyperprolactinemia, increased dietary lipids, and consumption of methylxanthines and caffeine may also contribute to the etiopathogenesis of mastalgia [4-6]. Psychoneurotic association has been found in various studies [7]. The assessment of mastalgia involves a detailed history including the severity, duration, and location and the relation of pain with the menstrual cycle, along with its impact on daily life. A thorough clinical examination of both breasts should be performed, to differentiate mammary from extramammary pain, and to rule out any lumps. Ultrasound or mammography (X-ray of the breast, taken in two views, cranio-caudal and medio-lateral oblique) are used as adjuncts to clinical examination [8].

Several treatment options have been used for mastalgia, but no particular guidelines are currently followed. In their study, Barros et al. suggested that reassuring mastalgia patients results in satisfaction of 70.2% (n=85) of patients once the possibility of cancer is ruled out [9]. Supportive brassiere may be especially beneficial for women with pendulous breasts to prevent overstretching of Cooper’s ligaments [4]. The various pharmacological treatment options include: (i) Evening primrose oil (EPO) along with vitamin E. Seeds of evening primrose contain large amounts of Ω-6 essential fatty acids-linoleic acid and γ-linoleic acid. Patients of mastalgia have low γ-linoleic acid levels. So, EPO will raise the levels and might relieve the patient of her pain. It has a direct action on immune cells of the body and indirect action on eicosanoids synthesis, which are raised significantly in patients of mastalgia; (ii) Non-steroidal anti-inflammatory drugs (NSAIDs): NSAIDs are a class of drugs that act by inhibiting cyclooxygenases (COX), which have a role in the formation of prostanoids from arachidonic acid. Hence, NSAIDs find their use as analgesics; (iii) Tamoxifen: selective estrogen receptor modulator (SERM); (iv) Danazol: androgenic hormonal drug; (v) Bromocriptine: dopamine receptor agonists; (vi) Ormeloxifene: SERM.

Psychological counseling and psychoeducation-relaxation therapy are reported to play an important and parallel part in the therapy [10]. Kinesiology (a form of therapy that uses muscle monitoring to look at imbalances that may be causing disease in the body) and acupuncture (point HT7) have also been used in various studies [10]. In this study, we performed the psychological assessment of patients presenting with mastalgia and also explored various treatment options available in terms of response to pain.

Due to the varying opinions prevalent in the literature regarding the psychomotor basis of mastalgia, the absence of any sufficient evidence regarding the association of treatment requirements with psychological status, and the absence of any particular treatment algorithm for mastalgia, this particular study was planned. The study aims to evaluate the psychological status and effectiveness of various treatment options in women presenting with mastalgia.

## Materials and methods

Study setting

This was a prospective comparative study. Consecutive consenting patients who presented to the General Surgery outpatient department of King George’s Medical University, Lucknow, India, with mastalgia from February 1 to November 30, 2021 (the duration of the study was 10 months) were included in this study. The entire group of patients were treated by the same team of surgeons. The data collection proforma is given in the Appendix.

Inclusion and exclusion criteria

Females of all age groups presenting to the General Surgery outpatient department with unilateral/ bilateral breast pain and/or chest wall pain were considered for this study. Pregnant patients (because our study included drugs that alter the hormonal status of the body, and hence may interfere with the course of pregnancy. Also, some of these drugs like tamoxifen are absolute contraindications in pregnancy), patients with a history of allergy to drugs (as continuing with such patients as a part of the study will be difficult in case of adverse drug reactions), or patients who were lost to follow-up were excluded from the study.

Procedures

Psychological Assessment

Each patient was assessed for her psychological status, on her first OPD visit, by the investigators in coordination with the consultant surgeon, using the Depression Anxiety and Stress Scale (DASS-42), a validated questionnaire to assess the negative emotional states of depression, anxiety, and stress status of a patient [11]. This particular scale was chosen as it allows objective assessment of the patient’s psychological status in a very short time (which can be done in an OPD setting), and the questions are easy to comprehend, already validated, and available in multiple languages (in our setting, the scale was made available in English and Hindi). The scores were evaluated for three parameters: Depression, Anxiety, and Stress, and were categorized (Table 1).

**Table 1:** DASS-42 Scale Source: Depression Anxiety and Stress Scale DASS (-42), Healthfocus Clinical Psychology Services, 2018 [11] DAS-42: Depression Anxiety and Stress Scale

	Normal	Mild	Moderate	Severe	Extremely Severe
Depression (D)	0-9	10-13	14-20	21-27	≥28
Anxiety (A)	0-7	8-9	10-14	15-19	≥20
Stress (S)	0-14	15-18	19-25	26-33	≥34

Clinical Examination

Evaluation of pain severity using the visual analog scale (VAS) score: A whole breast examination and a rollover test were performed for each patient. In a rollover test, the patient is asked to roll over to the opposite side, which allows the breast tissue to fall towards the midline by gravity resulting in flattening of the lateral half of the mammary gland. This helps to differentiate mammary pain from extramammary pain. The severity of pain was assessed using a VAS score. Participants were asked to score the pain they had experienced during the week before arrival at the OPD, from 1 (mild pain) to 10 (worst pain) and the VAS scores were evaluated by the investigators of the study in coordination with the consultant surgeon, during each OPD visit of the patient, and especially mandatorily in the first visit.

Other Investigations

Serum 25-OH vitamin D levels (Highly Sensitive Chemiluminescent Immunoassay), high-resolution ultrasound breasts (HRUSG) (Selenia® Dimensions® Mammography System; Hologic, Inc., Marlborough, Massachusetts, United States), and mammography obtained by (Affiniti 70 Ultrasound system; Koninklijke Philips N.V., Amsterdam, Netherlands) were obtained after their first OPD visit (only for women>35 years). Other investigations were based on the clinical presentation.

Categorization of patients

Patients complaining of breast pain might have either a mammary (breast pain) or an extra-mammary (chest wall pain) etiology. Even the breast wall pain may have multiple presentations like isolated breast(s) pain not associated with lumps (unremarkable physical examination), concurrent breast and chest wall pain, pain with a lump, or pain and tenderness only over the lump, with otherwise normal breast and chest wall. Treatment courses vary largely based on these clinical findings. So, based on clinical findings, the patients were differentiated into five categories. The first category comprised patients with isolated chest wall pain, like costochondritis, rib pain, and neck pain, which is referred to as the chest wall. The second category had patients with isolated breast(s) pain and the third category had patients with co-existing chest wall and breast(s) pain. Patients who had pain associated with a lump(s) were categorized into the fourth category, and the fifth group had patients with pain and tenderness over a lump with normal breast and chest wall (Table 2).

**Table 2:** Pain categories according to cause

Categories	Description
CAT-I	Patients with isolated chest wall pain (costochondritis, rib pain, neck pain referred to chest wall)
CAT-II	Patients with isolated breast(s) pain not associated with lumps (unremarkable physical examination)
CAT-III	Pain in both chest wall and breast(s)
CAT-IV	Pain and associated with lump/lumps.
CAT-V	Patients with pain and tenderness over lump with normal breast and chest wall

Management

Based on the VAS scores at the time of screening, patients were distributed into two groups: Group A (VAS ≤ 4 or pain not interfering with routine activities) and Group B (VAS > 4 or pain interfering with routine activities). Patients in Group A were reassured and asked to report if their pain did not subside. Patients in Group B underwent treatment and management as given below.

Treatment should be ideally escalated from non-hormonal to hormonal therapy, and from simpler to more complex options. Hence, the patients were started on either NSAIDs or EPO+vitamin E. Following this, they were started on hormonal options, beginning with tamoxifen (lesser side effects), followed by danazol (more side effects). Ormeloxifene is a comparatively newer option, so was kept at the last.

**Figure 1:**
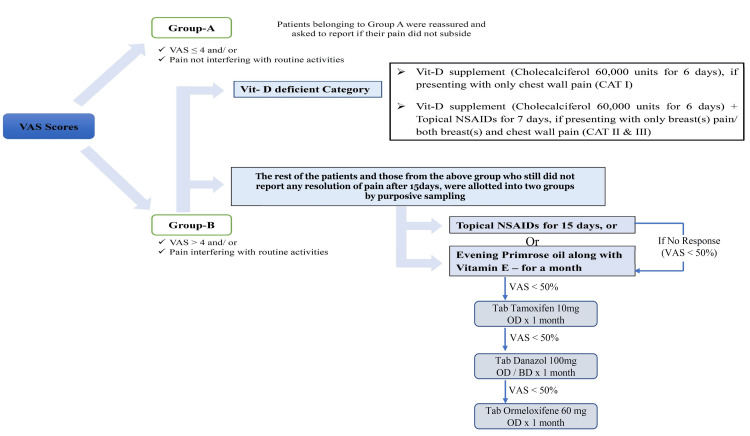
Flowchart of patient management algorithm followed in this trial VAS: visual analog scale; Vit: vitamin; CAT: category; NSAIDs: non-steroidal anti inflammatory drugs; OD: once a day Image credit: Authors

Patients of Group B were distributed into the topical NSAIDs group or EPO+Vitamin E group using the purposive sampling method. VAS scores of patients taking either of the medicines were re-evaluated and based on the scores, the patients were managed as shown in Figure 1.

The patients taking topical NSAIDs, who reported a decrease in VAS scores by ≥ 50% in a week were asked to continue the same medication for 15 days and were re-evaluated after two months. If the patient reported resolution of pain, they were considered successfully treated, else were prescribed EPO 1 gm thrice a day and vitamin E 400 mg once a day for a month and managed further. The patients on NSAIDs, who reported a decrease in VAS scores by < 50% were switched over to EPO+vitamin E for a month and managed further.

The patients on EPO+vitamin E who reported a decrease in VAS scores by ≥ 50% were asked to continue the same medications for two months and were re-evaluated after the two months. If the patient reported resolution of pain, they were considered successfully treated, or else were switched over to tablet tamoxifen 10 mg once a day for a month and managed further.

The patients on tamoxifen, who reported a decrease in VAS scores by ≥ 50% were asked to continue the same medication for two months and were re-evaluated after the two months. If the patient reported resolution of pain, they were considered successfully treated, or else were switched over to tablet danazol 100 mg once to twice a day for a month. The patients on danazol, who reported a decrease in VAS scores by ≥ 50% were asked to continue the same medication for two months and were re-evaluated after the two months. If the patient reported resolution of pain, they were considered successfully treated, else were switched over to the tablet ormeloxifene 30 mg twice a week for a month.

No treatment modality was continued for more than three months.

Ethical approval

The study was approved by the Institutional Ethics Committee of King George’s Medical University, Lucknow (ECR/262/Inst/UP/2013/RR-19) (reference number: 102ndECMIIA/P8). Written informed consent (in either English or Hindi) was taken from all patients before enrolling them in the study.

Statistical analysis

Statistical analysis was performed using IBM SPSS Statistics for Windows, Version 23.0 (Released 2015; IBM Corp., Armonk, New York, United States). All data was expressed as mean±SD. A non-parametric test (Chi-square test) was used to compare the DASS-42 category-wise score of the patients with their respective VAS scores at the first visit and duration of pain at the first visit. p-value<0.05 was considered significant. 

## Results

**Figure 2:**
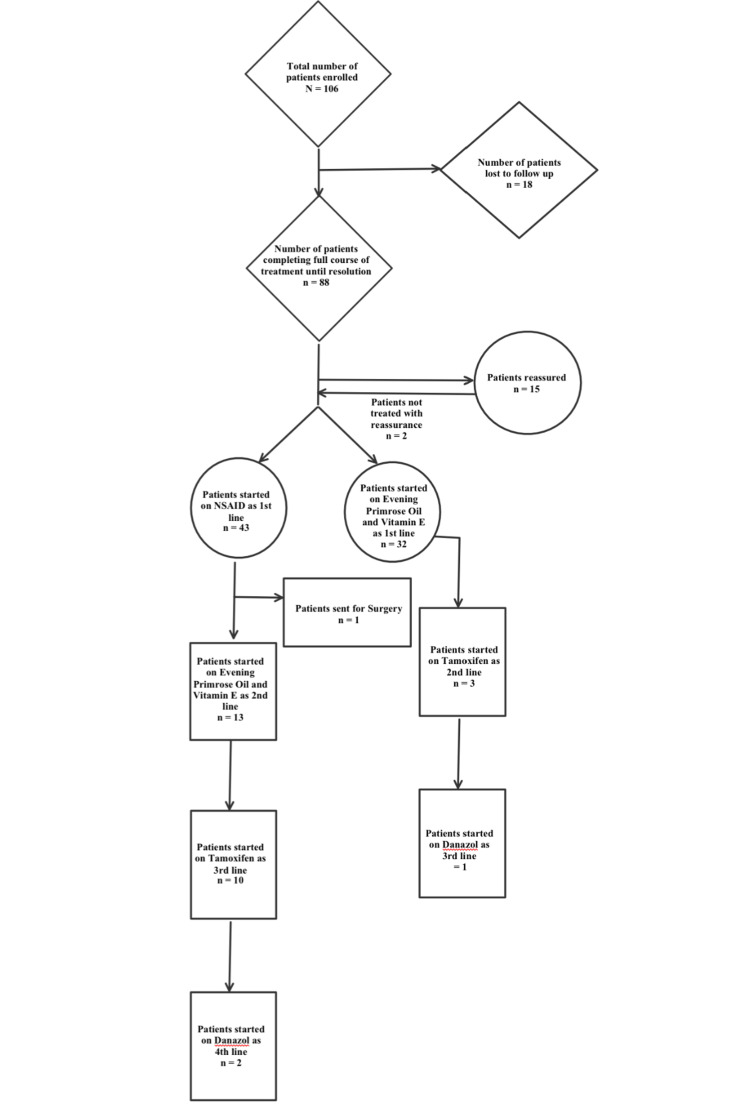
Flowchart of the number of patients treated at each stage.

A total number of 106 patients (all females) were enrolled in the study (Figure 2). The mean age of patients was 31.59 ± 10.52 years (range: 14-66 years). 

Clinical examination

Of the 106 patients, 82.08% (n = 87) had a cyclical pattern of pain, whereas 17.92% (n = 19) had a non-cyclical pattern of pain. Eighty-three (78.30%) patients presented with bilateral pain, whereas 14.15% (n = 15) had pain localized to the left side and 7.54% (n = 8) had right-sided pain. Out of 106 patients, 34.90% (n = 37) had isolated breast(s) pain, 14.15% (n = 15) had isolated chest wall pain, 50% (n = 53) had both breast(s) and chest wall pain and 0.94% (n = 1) had pain with an associated lump. The nature of pain was dull in 48.11% (n = 51) and pricking in nature in 21.70% (n = 23). The mean duration of pain of patients at the first visit was 70.13±18.29 weeks, which ranged from one week to 159 weeks. Four patients reported breast cancer in first-degree relatives (Table 3).

**Table 3:** Number of patients categorized on the basis of (A) cyclical or non-cyclical mastalgia, (B) laterality of pain, (C) pain in breast/chest wall/presence of lump, and (D) nature of pain (n=106) The data has been presented as number of patients (n) and percentage (%).

	Nature/Type of Pain	n (%)
A	Non-cyclical mastalgia	87 (82.08%)
	Cyclical mastalgia	19 (17.92%)
B	Bilateral pain	83 (78.30%)
	Unilateral left-sided pain	15 (14.15%)
	Unilateral right-sided pain	8 (7.54%)
C	Only breast(s) pain	37 (34.90%)
	Only chest wall pain	15 (14.15%)
	Both breast(s) and chest wall pain	53 (50.00%)
	Pain with lump	1 (0.94%)
	Pain over lump only	0
D	Constant	3 (2.83%)
	Dull	51 (48.11%)
	Pricking	23 (21.70%)
	Pinching	2 (1.89%)
	Tearing	6 (5.66%)
	Burning	8 (7.55%)
	Sharp	3 (2.83%)
	Stabbing	4 (3.77%)
	Diffuse	2 (1.89%)
	On pressure	2 (1.89%)
	Intermittent	2 (1.89%)

Psychological assessment

The average score for depression was 7.31 ± 8.53, for anxiety was 7.08 ± 6.57, and for stress was 11.15 ± 8.07. Seventy-seven (72.64%) patients had normal scores, 7.54% (n = 8) had mild, 10.38% (n = 11) had moderate, 3.77% (n = 4) had severe, and 5.66% (n = 6) had extremely severe scores for depression. For anxiety, 66.03% (n = 70) had scores in the normal range, 11.32% (n = 12) had mild scores, 10.38% (n = 11) had moderate scores, 5.66% (n = 6) had severe scores, and 6.60% (n = 7) had extremely severe scores. For stress, 66.98% (n = 71) had normal scores, 14.15% (n = 15) had mild scores, 13.20% (n = 14) had moderate scores, 4.71% (n = 5) had severe scores, and 0.94% (n = 1) had extremely severe scores (Table 4). 

**Table 4:** Distribution of patients based on their respective DASS-42 scores for depression, anxiety, and stress (into five categories: normal, mild, moderate, severe and extremely severe), and the respective r-values and p-values of DASS-42 scores with VAS score as well as with duration of pain at first visit (n=106) The data has been presented as number of patients (n) and percentage (%). p<0.05 has been considered as significant. *DASS-42 scores with VAS score at first visit; **DASS-42 scores with duration of pain at first visit DASS-42: Depression Anxiety and Stress Scale; VAS: visual analog scale

Grading		Depression	Anxiety	Stress
Normal		77 (72.64%)	70 (66.03%)	71 (66.98%)
Mild		8 (7.54%)	12 (11.32%)	15 (14.15%)
Moderate		11 (10.38%)	11 (10.38%)	14 (13.20%)
Severe		4 (3.77%)	6 (5.66%)	5 (4.71%)
Extremely Severe		6 (5.66%)	7 (6.60%)	1 (0.94%)
DASS-42 Score*	r-Value	-0.03	-0.001	-0.004
	p-Value	0.84	0.99	0.97
DASS-42 Score**	r-Value	0.04	-0.04	-0.02
	p-Value	0.69	0.66	0.85

Association of DASS-42 scores with VAS score at first visit

**Figure 3:**
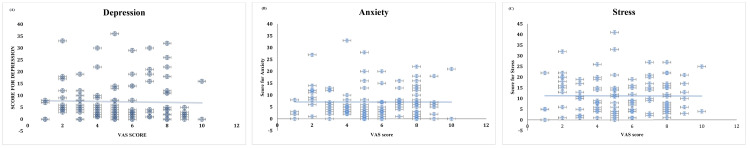
X-Y distribution curve of VAS Scores at first visit (x) with Depression (A), Anxiety (B), and Stress (C) scores (y), respectively VAS: visual analog scale

The bivariate correlation between each category of DASS-42 scores and the VAS score at the first visit was analyzed (Table 3). The correlation coefficient for depression, anxiety, and stress scores with VAS scores at the first visit is -0.03, (p =0.84), -0.001 (p=0.99), and -0.004 (p=0.97), respectively, implying that the scores had no significant correlation with the VAS scores at the first visit (p<0.05), which is also shown in the X-Y distribution curve in Figure 3.

Association of duration of pain at first visit with DASS-42 scores

**Figure 4:**
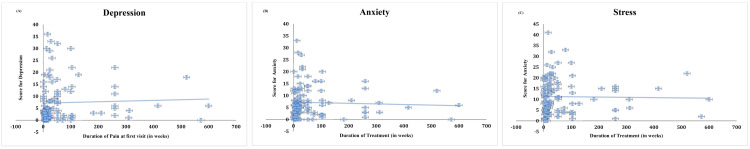
X-Y distribution curve of duration of pain at first visit (in weeks) (x) with Depression (A), Anxiety (B) and Stress (C) scores (y), respectively

We analyzed the bivariate correlation between each category of DASS-42 scores and the duration of pain at the first visit (Table 3). The correlation coefficient of depression, anxiety, and stress scores with the duration of pain at first were 0.04 (p=0.69), -0.04 (p=0.66), and -0.02 (p=0.85), respectively. This implies that the scores had no significant correlation with the duration of pain at the first visit (p<0.05), which can also be seen in the X-Y distribution curve in Figure 4.

Management

Out of 106, 88 (83.02%) patients completed the treatment until resolution. Of the 88 patients, 15 (17.05%) had a VAS score <4, and they were reassured without any treatment. On telephonic follow-up after seven days, the pain subsided in 13 (86.67%) patients, and two (13.33%) were started with further courses of treatment. Out of the remaining 75, 36 (48%) were given vitamin D supplementation, along with the treatment or as the sole treatment in case of only chest wall pain with associated vitamin D deficiency. Out of them, 15 (41.67%) had only chest wall pain. So, it was the sole treatment in them. All of them had resolution of their pain after the course of vitamin D. The rest 21 (58.33%) had been started on Vitamin D along with the other treatment options.

Forty-three patients were started with NSAIDs as the first line of treatment. Of them, 29 (67.44%) were completely treated, and one was operated for fibroadenoma. The rest 13 (30.23%) were moved to the second line of treatment, that is, EPO+vitamin E. Out of 13, three (23.07%) reported complete resolution. The rest 10 (76.92%) were started with the third line of treatment, tamoxifen 10 mg. The pain subsided completely in eight (80.00%) and the remaining two (20.00%) were switched over to danazol 100 mg, which was followed by complete resolution of pain subsequently.

Thirty-two patients were started on EPO+vitamin E as the first line of treatment. While 29 (90.63%) were completely relieved, three (10.34%) were switched to tamoxifen. While two (66.67%) of them were completely relieved, the remaining one was successfully treated using danazol. Menstrual irregularity was observed as a side-effect in one patient (33.33%) taking danazol for two months (Table 5).

**Table 5:** The percentage of patients who got treated in each step of treatment lines with respective average depression, anxiety, and stress scores The data has been presented as number (n) and percentage (%)*. * Percentage calculated out of total patients in the respective treatment groups.

Treatments Arms	Number of Patients and Percentage*		Average Score		
			Depression	Anxiety	Stress
NSAID as 1^st^ line	29/43	67.44%	7.76	7.41	11.48
NSAID Evening Primrose oil + Vitamin E	3/43	6.98%	9.38	8.00	12.44
NSAID, Evening Primrose oil + Vitamin E, Tamoxifen	8/43	18.60%	10.87	8.38	12.50
NSAID, Evening Primrose oil + Vitamin E, Tamoxifen, Danazol	2/43	4.65%	4.50	4.50	8.50
Evening Primrose oil + Vitamin E as 1^st^ line	29/32	90.63%	3.625	4.875	8.563
Evening Primrose oil + Vitamin E, Tamoxifen	2/32	6.25%	8	5.5	14
Evening Primrose oil + Vitamin E, Tamoxifen, Danazol	1/32	3.12%	9	7	20

Association of psychological status with treatment

**Figure 5:**
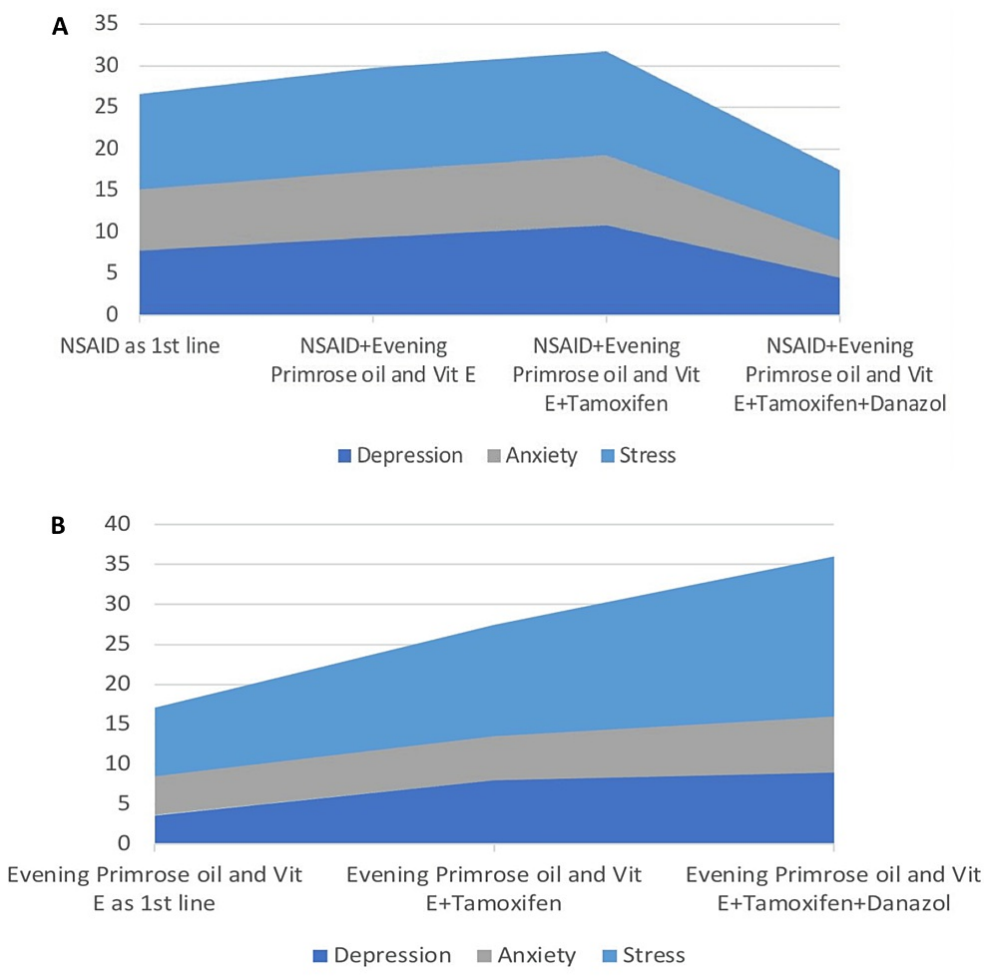
Average DASS-42 scores for patients who got treated at various lines of treatment: (A) patients who received NSAIDs as the first line of treatment, (B) patients who received EPO+Vitamin E as the first line of treatment DASS-42: Depression Anxiety and Stress Scale; NSAIDs: non-steroidal anti-inflammatory drugs; EPO: Evening Primrose Oil

Patients requiring a switchover to higher treatment options or who did not respond to initial lines of treatment had their initial DASS-42 scores higher than those who responded to initial lines of treatment (Figure 5, Table 4).

## Discussion

In the current study, the majority of patients, i.e. 87 (82.08%) had cyclical mastalgia, compared to the 19 (17.92%) who had non-cyclical mastalgia. Thirty-seven (34.90%) patients had isolated breast(s) pain, 15 (14.15%) had isolated chest wall pain, 53 (50%) had both breast(s) and chest wall pain, and one (0.94%) had pain with an associated lump. Our findings are in affirmation with the available literature, which also suggests cyclical mastalgia to be more common as compared to non-cyclical [12,13]. 

The psychological assessment of the patients elicited normal scores for depression anxiety and stress in the majority. The pain severity (VAS scores), and the duration of pain on the first visit were found to have no significant relationship with depression, anxiety, or stress levels (DASS-42 scores) (Figures 3-4). The results of our study do not correlate with similar studies, most of which state that the average psychological status in patients of mastalgia is worse than that of normal individuals and that mastalgia has a psychosomatic basis too. In a study conducted by Katar et al., the mean anxiety score (Beck Anxiety Inventory (BAI) scale used) and the mean depression score (Beck Depression Inventory (BDI) scale used) were higher (p<0.001) than the control group [13]. Studies by Aksu et al. [14], Colegrave et al. [15], Yilmaz et al. [16], Kanat BH et al. [17], and Preece et al. [18] also concluded similarly. On the contrary, similar to our study, a study by Cosar et al., patients with mastalgia had no difference in their depression scores as compared to the control group [19], where they used the Generalized Anxiety Disorder scale (GAD-7) for psychological assessment. All the above studies were conducted on non-Indian populations. Statistics related to breast health vary a lot in this part of the world, from that shown by Western data. Hence, assessing the Indian population is important, as both breast pathologies and psychosomatic conditions have shown a rising tendency in middle-income countries like India. Our study tried to do that, with the study population in the current study being mostly North Indians. The study results were quite different from the previous studies conducted on non-Indian populations.

For patients with low VAS scores (VAS< 4), reassurance and counseling proved to be beneficial, with 13 out of 15 (86.67%) reporting resolution. Reassurance and counseling were also found to be beneficial in a study by Barros et al. [20].

Twenty-nine of 43 patients (67.44%) who received topical NSAIDs as the first line of treatment, reported complete resolution of pain. In a study by Colak et al., follow-up after six months showed pain score in patients of cyclic mastalgia decreased by 5.87 ± 1.22 on NSAID administration as compared to the placebo group, which showed a very little decrease by 1.30 ± 1.34, and in patients with non-cyclic mastalgia, it decreased by 6.33 ± 1.34 on NSAID administration as compared to placebo group where it decreased by 1.12 ± 1.11 [21]. Also, a study by Irving et al. showed positive and rapid results with topical NSAIDs with no side effects reported [22]. Twenty-nine out of 32 (90.63%) patients reported completed resolution with only EPO+vitamin E, when started as the first line treatment, thus proving to be quite an efficient method of treatment. EPO has been shown to have variable benefits in various studies conducted over the years [12,23-30]. In patients in whom EPO+vitamin E was prescribed as second-line treatment after NSAID administration, only three out of 13 (23.08%) reported complete resolution of pain, indicating patients who are not resolved with NSAIDs also did not show much improvement to EPO+vitamin E. Such patients were switched over to tamoxifen as the next line of treatment. 

Eight out of 43 (18.60%) of the NSAID arm and two out of 32 (6.25%) of the EPO+vitamin E arm were completely relieved of their pain using tamoxifen, thus showing an 80% (eight out of 10) success rate in cases resistant to both NSAIDs and EPO and 66.67% (two out of three) success rate in cases resistant to EPO. We did not observe any side effects of tamoxifen in our study as it was administered for a short term of ≤ three months. 

The two non-responders (4.65% of the NSAID arm) to tamoxifen from topical NSAIDs as the first line treatment group and the one (3.12% of the EPO+vitamin E arm) from the evening primrose oil plus vitamin E group, were successfully relieved of pain using danazol. Danazol is far more effective than a placebo in several studies [31,32]. In another study comparing tamoxifen, danazol, and placebo, both tamoxifen and danazol were found to be similar in effectiveness (72% and 65%, respectively), and the placebo reported 38% effectiveness [33]. Menstrual irregularity was observed as a side-effect in one patient (33.33%) taking danazol for two months. 

Pain in all the patients subsided by initial lines of treatment, hence no patients were switched over to ormeloxifene.

We did not find a psychoneurotic basis for mastalgia in our study. The DASS-42 scores had no significant correlation with pain or the duration of pain at the initial visit. But, an indirect indication of the psychoneurotic basis of mastalgia can be seen in Figure 5, where it can be observed that patients who required further upgradation in treatment lines were also the ones having worse DASS-42 scores (higher scores). However, generalization of this fact will require further studies to be conducted on these lines.

Strengths and limitations of the study

Our study attempts to formulate an algorithm-based approach for patients with mastalgia, which has been largely lacking, despite abundant treatment options available. This study also emphasizes that the majority of women can be treated with reassurance, topical NSAIDs, and EPO, while the use of hormonal treatment can be reserved for a selected few.

The current study used a purposive sampling method; hence, the possibility of selection bias can't be entirely ruled out. Also, the results can't be generalized, as all patients were from the northern part of India. Further studies on different study populations are essential to generalize these results. The number of patients requiring tamoxifen and danazol was extremely low in both groups and hence the role of these drugs couldn't be studied extensively. Ormeloxifene, another popular treatment option for mastalgia, couldn’t be tested in our study since all patients resolved by earlier lines of treatment. Also, our study didn’t explore the options of formulation of a treatment algorithm that can be safe at all steps for pregnant women and for those who were allergic to drugs, due to their exclusion from the study population.

Future studies

The association of psychological status with treatment lines couldn't be generalized in our study due to the fewer number of patients in the higher treatment lines, which in turn was due to the algorithmic design of this study. So, options for studies with different designs and higher sample sizes should be explored further. Moreover, further studies are required on other groups of the population to generalize the results. Even within India, populations in the different regions vary a lot in characteristics. For example, South India is inhabited mostly by Dravidian population while North India is primarily inhabited by Indo-Aryan population. Hence, even for national algorithms to be in place, further studies are essential. Also, our study couldn't elicit a clear-cut relation between psychological status and mastalgia; hence, further studies with larger sample sizes are required to be conducted. To probe into the possible cause behind this, more studies are required to be conducted. Exploration of the options for the formulation of a treatment algorithm that can be safe at all levels for pregnant women and individuals who are allergic to drugs should be done in the future, with the collaboration of obstetricians and pharmacologists.

## Conclusions

Differentiating mammary from extramammary pain is important before initiating any treatment in patients with mastalgia. Reassurance and counseling, along with lifestyle modifications, appropriate diet, and adequate breast support are sufficient in the majority of patients with low VAS scores. Most of the patients were relieved of mastalgia with NSAIDs or EPO and vitamin E in our study, with very few requiring second-line hormonal treatment like tamoxifen and danazol. The algorithm proposed by us, which included starting with non-hormonal simpler pharmacotherapies like NSAIDs or EPO and vitamin E (supplemented by vitamin D supplementation in case of its deficiency), followed by hormonal options like tamoxifen (fewer side-effects) succeeded by danazol (more side-effects), proved to be quite successful in treating patients, with complication noted in just one out of 88 patients. The duration or severity of pain did not correlate with the psychological condition of the patient, but this doesn’t rule out the importance of psychological evaluation in patients of mastalgia, because it was also observed in our study that patients who required further upgradation in treatment lines, were also the ones having worse DASS-42 scores.

## Figures and Tables

**Figure 6:**
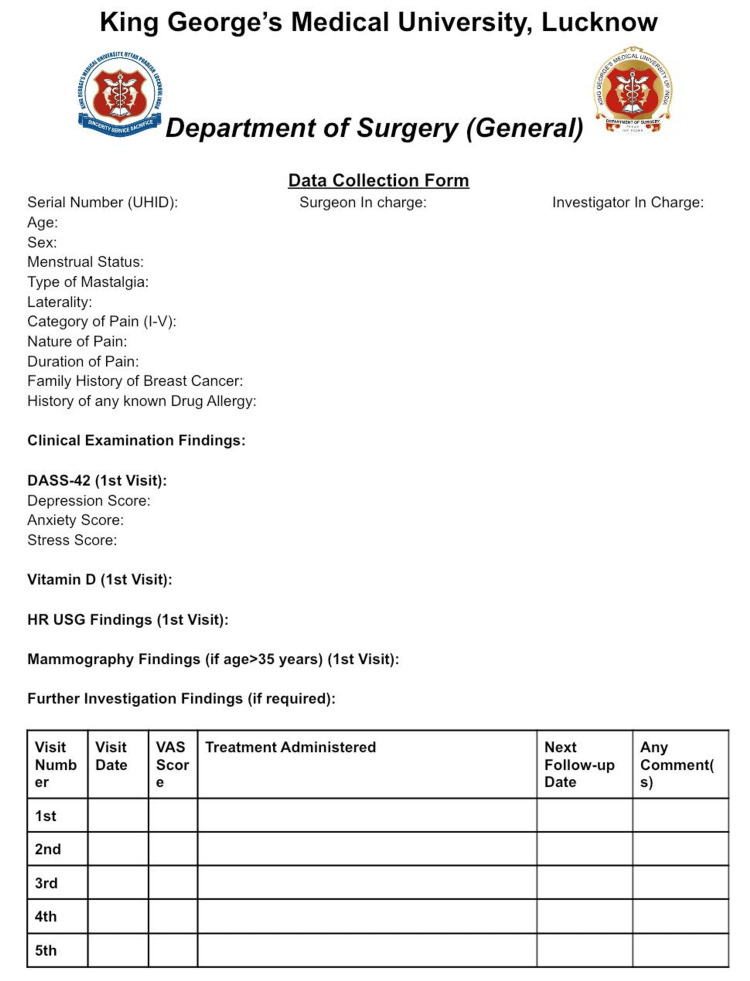
Patient data collection proforma

## Appendices

Data collection proforma
